# Hepatitis B vaccination timing: results from demographic health surveys in 47 countries

**DOI:** 10.2471/BLT.16.178822

**Published:** 2017-01-26

**Authors:** Aparna Schweitzer, Manas K Akmatov, Gérard Krause

**Affiliations:** a Helmholtz Centre for Infection Research (HZI), Inhoffenstraße 7, Brunswick 38124, Germany.

## Abstract

**Objective:**

To examine the impact of hepatitis B vaccination schedules and types of vaccines on hepatitis B vaccination timing.

**Methods:**

We used data for 211 643 children from demographic and health surveys in 47 low- and middle-income countries (median study year 2012). Data were from vaccination cards and maternal interviews. We grouped countries according to the vaccination schedule and type of vaccine used (monovalent or combination). For each country, we calculated hepatitis B vaccination coverage and timely receipt of vaccine doses. We used multivariable logistic regression models to study the effect of vaccination schedules and types on vaccination delay.

**Findings:**

Substantial delays in vaccination were observed even in countries with fairly high coverage of all doses. Median delay was 1.0 week (interquartile range, IQR: 0.3 to 3.6) for the first dose (*n* = 108 626 children) and 3.7 weeks (IQR: 1.4 to 9.3) for the third dose (*n* = 101  542). We observed a tendency of lower odds of delays in vaccination schedules starting at 6 and at 9 weeks of age. For the first vaccine dose, we recorded lower odds of delays for combination vaccines than for monovalent vaccines (adjusted odds ratio, aOR: 0.76, 95% confidence interval, CI: 0.71 to 0.81).

**Conclusion:**

Wide variations in hepatitis B vaccination coverage and adherence to vaccination schedules across countries underscore the continued need to strengthen national immunization systems. Timely initiation of the vaccination process might lead to timely receipt of successive doses and improved overall coverage. We suggest incorporating vaccination timing as a performance indicator of vaccination programmes to complement coverage metrics.

## Résumé

**Objectif:**

Étudier l'impact des calendriers de vaccination et des types de vaccins contre l'hépatite B sur la date des vaccinations contre l'hépatite B.

**Méthodes:**

Nous avons eu recours à des données concernant 211 643 enfants qui étaient issues d'enquêtes démographiques et sanitaires menées dans 47 pays à revenu faible et intermédiaire (année médiane: 2012). Ces données provenaient de carnets de vaccination et d'entretiens avec les mères. Nous avons regroupé les pays en fonction du calendrier de vaccination et du type de vaccin utilisé (monovalent ou combiné). Pour chaque pays, nous avons calculé la couverture vaccinale contre l'hépatite B ainsi que l'administration en temps voulu des doses du vaccin. Nous avons utilisé des modèles de régression logistique multivariée pour étudier l'effet des calendriers de vaccination et des types de vaccins sur les retards de vaccination.

**Résultats:**

D'importants retards de vaccination ont été observés, y compris dans les pays où la couverture vaccinale était relativement élevée, pour toutes les doses. Le retard moyen était de 1,0 semaine (intervalle interquartile, IQR: 0,3 à 3,6) pour la première dose (*n* = 108 626 enfants) et de 3,7 semaines (IQR: 1,4 à 9,3) pour la troisième dose (*n* = 101 542). Nous avons observé que la probabilité de retards avait tendance à être plus faible pour les calendriers de vaccination qui débutaient à l'âge de 6 et de 9 semaines. Pour la première dose vaccinale, nous avons noté une probabilité de retard plus faible lorsqu'il s'agissait de vaccins combinés que de vaccins monovalents (rapport des cotes ajusté: 0,76, intervalle de confiance de 95%: 0,71 à 0,81).

**Conclusion:**

Les écarts importants au niveau de la couverture vaccinale contre l'hépatite B et du respect des calendriers de vaccination dans les différents pays soulignent la nécessité de continuer à renforcer les systèmes nationaux de vaccination. Débuter les vaccinations en temps voulu pourrait permettre d'administrer les rappels en temps voulu également et d'améliorer la couverture globale. Nous suggérons d'intégrer la date des vaccinations comme indicateur de performance des programmes de vaccination, en complément de la mesure de la couverture vaccinale.

## Resumen

**Objetivo:**

Examinar el impacto de los calendarios de vacunación de la hepatitis B y los tipos de vacunas en los plazos de vacunación de la hepatitis B.

**Métodos:**

Se utilizaron datos de 211 643 niños de encuestas demográficas y de salud en 47 países con ingresos bajos y medios (año promedio de estudio 2012). La información provenía de las tarjetas de vacunación y de entrevistas a las madres. Se agruparon los países según el calendario de vacunación y el tipo de vacuna utilizada (monovalente o combinada). Para cada país, se calculó una cobertura de vacunación contra la hepatitis B y la recepción oportuna de las dosis de la vacuna. Se utilizaron modelos de regresión logística multivariable para estudiar el efecto de los calendarios de vacunación y los tipos en el retraso en la administración de vacunas.

**Resultados:**

Se observaron grandes retrasos en la vacunación, incluso en países con una cobertura bastante alta de todas las dosis. El retraso medio era de 1,0 semanas (rango intercuartílico, ICR: 0,3 a 3,6) para la primera dosis (*n* = 108 626 niños) y de 3,7 semanas (ICR: 1,4 a 9,3) para la tercera dosis (*n* = 101 542). Se observó una tendencia de menores probabilidades de retraso en los calendarios de vacunación que empezaban a las 6 y 9 semanas de edad. Para la primera dosis de la vacuna, se registraron menos probabilidades de retraso para las vacunas combinadas que para las monovalentes (coeficiente de posibilidades ajustado: 0,76, intervalo de confianza (IC) del 95%: 0,71 a 0,81).

**Conclusión:**

Las grandes diferencias en la cobertura de vacunación contra la hepatitis B y la adherencia a los calendarios de vacunación entre países destacan la continua necesidad de mejorar los sistemas nacionales de inmunización. La iniciación oportuna del proceso de vacunación puede dar lugar a la recepción oportuna de dosis sucesivas y a la mejora de la cobertura general. Sugerimos la incorporación de la fecha de vacunación como un indicador de rendimiento de los programas de vacunación para completar el cálculo de la cobertura.

## ملخص

**الغرض:**

فحص تأثير جداول التحصين ضد فيروس التهاب الكبد (ب) وأنواع التطعيمات على توقيت التحصين ضد التهاب الكبد (ب).

**الطريقة:**

استخدمنا البيانات الخاصة بـ 211643 طفلاً من المسوح الديموغرافية والصحية في 47 بلدًا من البلدان ذات الدخل المنخفض والمتوسط (دراسة متوسطة لعام 2012). وتم استخراج البيانات من واقع بطاقات التحصين والمقابلات التي أُجريت مع الأمهات. وقمنا بتجميع البلدان وفقًا لجدول التحصين ونوع التطعيم المستخدم (أحادي التكافؤ أم مُركّب). وقمنا بحساب تغطية التحصين ضد فيروس التهاب الكبد (ب) وتلقي جرعات التحصين المناسبة لكل بلد. ثم استخدمنا نماذج تحوف لوجيستية متعددة المتغيرات لدراسة تأثير جداول التحصين وأنواعه على تأخير التطعيم.

**النتائج:**

تم ملاحظة تأخيرات كبيرة في التحصين حتى في البلدان التي بها تغطية عالية إلى حد ما لجميع الجرعات. وكان متوسط التأخير 1.0 أسبوع (المدى الربيعي: من 0.3 إلى 3.6) للجرعة الأولى (العدد = 108626 طفل و3.7 أسبوع (المدى الربيعي: من 1.4 إلى 9.3) للجرعة الثالثة (العدد = 101542). وقد لاحظنا ميلاً لانخفاض احتمالية التأخير في جداول التحصين بدءًا من عمر 6 و9 أسابيع. بالنسبة للجرعة الأولى من التطعيم، سجلنا انخفاض احتمالية التأخير للتطعيمات المركبة أكثر من التطعيمات أحادية التكافؤ (نسبة الاحتمالات المعُدلة: 0.76، وبنسبة أرجحية مقدارها 95‏%: 0.71 إلى 0.81).

**الاستنتاج:**

أشارت الاختلافات الكبيرة في تغطية التحصين ضد فيروس التهاب الكبد (ب) والالتزام بجداول التحصين عبر البلدان إلى الحاجة المستمرة لتعزيز أنظمة التحصين. وقد يؤدي البدء في الوقت المناسب لعملية التحصين إلى تلقي الجرعات المتتالية والتغطية الشاملة المحسنة في الوقت المناسب. ونقترح إدراج وقت التحصين كمؤشر لأداء برامج التحصين لاستكمال مقاييس التغطية.

## 摘要

**目的:**

旨在调查乙肝疫苗接种程序表和疫苗类型对乙肝疫苗接种时间的影响。

**方法:**

我们采纳了在 47 个中低收入国家开展的人口与健康调查中的 211 643 名儿童的调查数据（调查年份中值为 2012）。 数据来源于疫苗接种卡和对母亲的访谈。 我们根据接种程序表和疫苗类型（单价疫苗或联合疫苗）对国家进行了分组。 计算出各个国家乙肝疫苗接种覆盖率和接种各剂疫苗的及时性。 并且采用多变量逻辑回归模型研究了疫苗接种程序表和疫苗类型对接种延迟的影响。

**结果:**

我们观察到，即使是在各剂疫苗覆盖率均相当高的国家，依然有很多延迟接种的情况。 第一剂 （*n* = 108 626 名儿童）的延迟时长中值是 1.0 周（四分位差 IQR：0.3 到 3.6）；第三剂 (*n* = 101  542) 是 3.7 周（IQR：1.4 到 9.3）。 我们还观察到，从年龄为 6 周和 9 周开始的接种程序表的延迟概率呈降低趋势。 记录显示，对于第一剂疫苗，联合疫苗的延迟概率比单价疫苗的延迟概率更低（调整过的比值比： 0.76，95% 置信区间： 0.71 到 0.81）

**结论:**

不同国家在乙肝疫苗覆盖率和遵循接种程序表方面的较大差异突出了持续加强国家免疫系统的需求。 及时开始疫苗接种可能会有助于及时连续地接种各剂疫苗和提升整体覆盖率。 我们建议将疫苗接种时间纳入疫苗接种计划的绩效指标，以补充覆盖率衡量标准。

## Резюме

**Цель:**

Изучить влияние графиков вакцинации против гепатита В и типов вакцин на сроки вакцинации против гепатита В.

**Методы:**

Авторы использовали данные по 211 643 детям, участвовавшим в демографических и медико-санитарных обследованиях, в 47 странах с низким и средним уровнем дохода (медиана лет исследований — 2012 год). Данные были получены из карт вакцинации и опросов матерей. Страны были распределены по группам в зависимости от графика вакцинации и типа используемой вакцины (моновалентная или комбинированная). Для каждой страны был рассчитан охват вакцинацией против гепатита В и определена своевременность получения доз вакцины. С помощью моделей множественной логистической регрессии было изучено влияние графиков вакцинации и типов вакцин на задержку в проведении вакцинации.

**Результаты:**

Значительные задержки в проведении вакцинации наблюдались даже в странах с достаточно высоким уровнем охвата всеми дозами. Средняя продолжительность задержки составила 1,0 недели (межквартильный размах, МКР: от 0,3 до 3,6) для первой дозы (*n* = 108 626 детей) и 3,7 недели (МКР: от 1,4 до 9,3) для третьей дозы (*n* = 101 542). Наблюдалась тенденция к снижению вероятности задержки в случае с графиками, предполагающими начало вакцинации на 6-й и 9-й неделе рекомендованного возраста. Вероятность задержки в получении первой дозы вакцины при применении комбинированных вакцин была ниже, чем при применении моновалентных вакцин (скорректированное отношение шансов: 0,76; 95%-й доверительный интервал: от 0,71 до 0,81).

**Вывод:**

Значительная разница в охвате вакцинацией против гепатита В и соблюдении графиков вакцинации в разных странах подчеркивает сохраняющуюся потребность в укреплении национальных систем иммунизации. Своевременное начало процесса вакцинации может привести к своевременному получению последующих доз и улучшению общего охвата. Авторы рекомендуют включить сроки вакцинации в качестве показателя эффективности в программы вакцинации в дополнение к показателям охвата.

## Introduction

Chronic hepatitis B virus (HBV) infection continues to make a substantial contribution to the global burden of disease.^1^^,^^2^ The risk of developing chronic HBV is inversely related to the age at acquisition of infection.^3^^,^^4^ Immunization is the most effective measure to prevent the transmission of HBV.^5^^,^^6^ In 2014, the World Health Organization (WHO) reaffirmed the need for hepatitis B vaccines to become an integral part of national immunization schedules.^7^ WHO recommends a birth dose within 24 hours of birth to prevent perinatal and early horizontal HBV transmission.^8^ The birth dose should be followed by 2 or 3 doses of monovalent or multivalent hepatitis B vaccines.^8^

Vaccination coverage estimates from WHO and the United Nations Children’s Fund (UNICEF) capture the proportion of vaccinated children in specific age groups. However, these estimates provide little insight into the extent to which vaccinations are administered on time and they tend to understate the susceptibility to HBV infection in a population.^9^^–^^11^ In practice, vaccinations are more likely to be received late than early.^12^^,^^13^ When hepatitis B vaccination is delayed, children fail to receive adequate protection when they are most vulnerable. Moreover, by increasing the period of susceptibility to infection,^8^ late vaccinations raise the risk of HBV infection^14^ and hence the risk of chronicity. Furthermore, a delay in one dose may lead to delays in further doses,^15^ thereby extending the at-risk period. This has important implications in countries that are highly endemic for HBV infection. In this situation, catch-up vaccination of older children has relatively little impact because they might already be infected by the time they present for vaccination.^8^

There are multiple options for incorporating hepatitis B vaccines into national immunization programmes and the choice of vaccination schedule depends primarily on programmatic considerations.^8^ From a policy perspective, data from a large number of countries are necessary to evaluate the impact of existing hepatitis B vaccination schedules and vaccine types on hepatitis B vaccination timing. Thus far, analyses of hepatitis B vaccinations have been limited in scope^16^^–^^18^ and have not tackled this aspect. The demographic and health surveys (DHS) provide data on childhood vaccinations based on vaccination cards and maternal interviews. Data compiled through DHS are nationally representative and are considered to be the best available data on vaccination coverage.^19^ We estimated vaccination coverage and timing, and examined the impact of hepatitis B vaccination schedules and vaccine types on vaccination timing in countries for which DHS data were publicly available.

## Methods

### Study design

Full details of DHS methods have been reported elsewhere.^20^^,^^21^ DHS data on hepatitis B vaccination were available for 54 countries. For every country, we used the most recent survey available until the end of 2015. Seven surveys were excluded due to incomplete data or non-standard recording of dates. We therefore included 47 countries with survey years ranging from 2005 to 2014. We grouped countries based on their vaccination schedule and type of vaccine (monovalent or combination) in use (Table 1, available at http://www.who.int/bulletin/volumes/95/3/16.178822). In countries that had altered their schedules before the DHS survey we limited our analyses to the more established vaccination schedule.

**Table 1:** Background characteristics and sampling for the 47 low- and middle-income countries surveyed, by national hepatitis B vaccination schedule

Vaccination schedule^a^ and vaccine type	Country	WHO Region	Country data				DHS survey year	Sample of children aged 12–60 months, no.^f^
			Gavi financing^b^	Income level^c^	Population^d^	HBsAg prevalence, (%)^e^		
**Weeks 0, 4, 13**								
Monovalent	Maldives	SEAR	No	Upper-middle	332 575	N/A	2009	2 498
**Weeks 0, 4, 26**								
Monovalent	Republic of Moldova	EUR	No	Lower-middle	3 573 024	7.4	2005	1 165
**Weeks 0, 6, 14**								
Monovalent	Nigeria	AFR	No	Lower-middle	159 707 780	9.8	2013	20 799
**Weeks 0, 6, 26**								
Monovalent	Armenia	EUR	Yes	Lower-middle	2 963 496	N/A	2010	1 114
**Weeks 0, 9, 17**								
Monovalent	Azerbaijan	EUR	Yes	Upper-middle	9 094 718	2.8	2006	1 707
Monovalent	Tajikistan	EUR	Yes	Lower-middle	7 627 326	7.2	2012	3 797
**Weeks 0, 9, 22**								
Monovalent	Kyrgyzstan	EUR	Yes	Lower-middle	5 334 223	10.3	2012	3 174
**Weeks 0, 9, 26**								
Monovalent	Albania	EUR	Yes	Upper-middle	3 150 143	7.8	2008	1 303
**Weeks 4, 8, 12**								
Tetravalent	United Republic of Tanzania	AFR	Yes	Low	44 973 330	7.2	2010	5 444
Pentavalent	Uganda	AFR	Yes	Low	33 987 213	9.2	2011	1 586
**Weeks 6, 10, 14**								
Monovalent	Bangladesh	SEAR	Yes	Lower-middle	151 125 475	3.1	2011	6 400
Monovalent	Cameroon	AFR	Yes	Lower-middle	20 624 343	12.2	2011	3 803
Monovalent	Gabon	AFR	No	Upper-middle	1 556 222	11.5	2012	2 605
Monovalent	Lesotho	AFR	Yes	Lower-middle	2 010 586	N/A	2009	1 263
Monovalent	Pakistan	EMR	Yes	Lower-middle	173 149 306	2.8	2012	2 865
Monovalent	Swaziland	AFR	No	Lower-middle	1 193 148	19.0	2006	1 610
Monovalent	Timor-Leste	SEAR	No	Lower-middle	1 057 122	N/A	2009	7 168
Bivalent	Benin	AFR	Yes	Low	9 509 798	15.6	2011	6 571
Tetravalent	Madagascar	AFR	Yes	Low	21 079 532	4.6	2008	4 269
Tetravalent	Mozambique	AFR	Yes	Low	23 967 265	8.3	2011	7 412
Pentavalent	Burundi	AFR	Yes	Low	9 232 753	9.1	2010	2 625
Pentavalent	Cambodia^g^	WPR	Yes	Lower-middle	14 364 931	4.1	2014	3 487
Pentavalent	Comoros	AFR	Yes	Low	698 695	N/A	2012	2 100
Pentavalent	Côte d’Ivoire	AFR	Yes	Lower-middle	18 976 588	9.4	2011	2 383
Pentavalent	Democratic Republic of the Congo	AFR	Yes	Low	62 191 161	6.0	2013	6 462
Pentavalent	Ghana	AFR	Yes	Lower-middle	24 262 901	12.9	2014	2 103
Pentavalent	Kenya	AFR	Yes	Lower-middle	40 909 194	5.2	2008	3 965
Pentavalent	Liberia	AFR	Yes	Low	3 957 990	17.6	2013	2 469
Pentavalent	Malawi	AFR	Yes	Low	15 013 694	12.2	2010	3 945
Pentavalent	Mali	AFR	Yes	Low	13 985 961	13.1	2012	3 700
Pentavalent	Namibia	AFR	No	Upper-middle	2 178 967	8.6	2013	1 357
Pentavalent	Niger	AFR	Yes	Low	15 893 746	15.5	2012	2 282
Pentavalent	Rwanda	AFR	Yes	Low	10 836 732	6.7	2010	3 259
Pentavalent	Senegal	AFR	Yes	Low	12 950 564	11.1	2014	4 246
Pentavalent	Sierra Leone^g^	AFR	Yes	Low	5 751 976	8.4	2013	3 606
Pentavalent	Zambia	AFR	Yes	Lower-middle	13 216 985	6.1	2013	9 562
**Weeks 9, 13, 17**								
Monovalent	Jordan	EMR	No	Upper-middle	6 454 554	1.9	2012	5 380
Pentavalent	Burkina Faso	AFR	Yes	Low	15 540 284	12.1	2010	5 113
Pentavalent	Congo	AFR	Yes	Lower-middle	4 111 715	11.0	2011	3 508
**Weeks 9, 17, 26**								
Monovalent	Egypt	EMR	No	Lower-middle	78 075 705	1.7	2014	11 639
Monovalent	Colombia^g^	AMR	No	Upper-middle	46 444 798	2.3	2010	12 615
Pentavalent	Bolivia (Plurinational State of)	AMR	No	Lower-middle	10 156 601	0.4	2008	6 396
Pentavalent	Dominican Republic^g^	AMR	No	Upper-middle	10 016 797	4.1	2013	2 597
Pentavalent	Guyana	AMR	Yes	Upper-middle	753 362	N/A	2009	1 449
Pentavalent	Honduras	AMR	No	Lower-middle	7 503 875	N/A	2011	7 998
Pentavalent	Peru^g^	AMR	No	Upper-middle	29 262 830	2.1	2012	7 513
**Weeks 13, 17, 22**								
Pentavalent	Zimbabwe	AFR	Yes	Low	13 076 978	14.4	2010	3 331
**Overall**	N/A	N/A	N/A	N/A	1 161 836 962	N/A	N/A	211 643

AFR: African Region; AMR: Region of the Americas; DHS: Demographic Health Survey; EMR: Eastern Mediterranean Region; EUR: European Region; Gavi: Gavi, the Vaccine Alliance; HBsAg: surface antigen of the hepatitis B virus; N/A: data not available or not applicable; SEAR: South-East Asia Region; WPR: Western Pacific Region; WHO: World Health Organization.

^a^ Schedule is the target weeks after birth to administer the first, second and third doses of vaccine. Details of national immunization schedules were obtained from relevant annual joint World Health Organization (WHO) and United Nations Children’s Fund (UNICEF) immunization reports and demographic and health surveys for each country. Vaccine types were: monovalent (hepatitis B); bivalent (hepatitis B and *Haemophilus influenzae* type b); tetravalent (hepatitis B and diphtheria–tetanus–pertussis); pentavalent (diphtheria–tetanus–pertussis, hepatitis B and *Haemophilus influenzae* type b).

^b^ Gavi financing was recorded as “Yes” if the country received new and underused vaccine support for either monovalent or pentavalent vaccines (http://www.gavi.org/country/).

^c^ Country income level was defined as per the World Bank.^22^

^d^ Population estimates were obtained from the United Nations.^23^

^e^ Data on HBsAg prevalence (general population aged 0–85 years) are the most recent global prevalence estimates from 1965–2014 obtained from Schweitzer et al.^2^

^f^ Sample sizes (number of children aged 12‒60 months) are unweighted.

^g^ Vaccination schedule in these countries includes a birth dose of hepatitis B vaccine (monovalent), i.e. four doses in total.

Notes: We examined data quality for all children covered by the surveys. Vaccination dates were counted as invalid if day, month or year were missing, or if the date was implausible, e.g. before the date of birth of the child or after the date of mother’s interview or with erroneous dates (e.g. as year 9998). We only considered vaccination cards as available if seen by the interviewer. Excluded surveys: Ethiopia (non-standard date recording), Indonesia (date of birth not available), Morocco (only first dose reported), Nepal (non-standard date recording), Nicaragua (key missing variables, e.g. wealth index), Philippines (date of birth not available),and Turkey (date of birth not available). Countries that altered their national immunization schedules within 5 years of the survey were: Armenia (pentavalent introduced in 2009), Gabon (pentavalent introduced in 2010), Kyrgyzstan (pentavalent introduced in 2009) and Tajikistan (pentavalent introduced in 2008–09). Hence, we adopted the previous immunization schedule for these nations in our analysis. For Cambodia and Colombia, and the United Republic of Tanzania, data on multiple vaccine types (monovalent and combination) were reported. We based our estimates on monovalent vaccination in Colombia, pentavalent in Cambodia and tetravalent in the United Republic of Tanzania. The decision was based on schedules (vaccines) reported in the relevant annual UNICEF/WHO immunization reports and the available data sets.

We identified and analysed individual vaccine doses according to the respective country’s national immunization schedule. To assess vaccination coverage, we used only documented vaccinations (with or without specific dates marked) for each vaccine dose. Vaccination coverage was categorized as complete if the child was recorded as fully immunized with three or four doses of the vaccine according to the country’s national immunization schedule. Vaccination coverage was categorized as incomplete if any of the recommended doses were recorded as 0 (not given), including when data on other doses was missing.^8^ We excluded children younger than 12 months to avoid the drawback of censored observations. The denominator for coverage was the DHS sample of surviving children born in the past 5 years before the survey (or sometimes 3 years, depending on the DHS interval). To address potential bias from maternal recall,^24^^,^^25^ we estimated crude vaccination coverage and completeness (from vaccination card plus maternal recall).

To assess vaccination timing, we compared each child’s recorded vaccination dates with those recommended in the country’s national immunization schedule. Age at vaccination was determined by subtracting the child’s date of birth from valid vaccination dates. Vaccinations were categorized as timely if administered within 4 weeks of the recommended age, or delayed if administered more than 4 weeks after the recommended age. We calculated the percentage of children receiving delayed or timely vaccinations. The denominator for calculating timing included children vaccinated early, i.e. before the recommended age. National immunization schedules often do not specify when to give the birth-dose vaccine.^26^ We therefore defined a timely birth dose as received within 7 days after delivery, based on the evidence on effective prevention of perinatal hepatitis B transmission.^27^ We also computed estimates based on the WHO recommendation of giving hepatitis B vaccine within 24 hours of birth.^8^

### Statistical analysis

We performed all analyses with the survey functions of Stata statistical software, version 14 (Stata Corp., College Station, United States of America), using a significance level of ≤ 0.05.

We took account of the complex DHS survey design and used sample weights provided in the available data sets. Using Spearman rank correlations, we analysed the relationship between vaccination timing and coverage of the third dose of vaccine across countries.

We then used binary multivariable logistic regression models to calculate adjusted odds ratios (aOR) and 95% confidence intervals (CI) to investigate the impact of vaccination schedule and vaccine type on hepatitis B vaccination timing. Vaccinations were dichotomized as delayed or timely. We constructed pooled models for two outcomes: delayed first dose and delayed third dose. The main independent variables were the recommended week of the vaccination schedule and vaccine type (monovalent or combination). We categorized reported vaccination schedules as follows: starting at birth i.e. ≤ 1 week of age (reference category), 4, 6, 9 and 13 weeks, respectively. We incorporated covariates chosen for their possible or demonstrated associations with vaccination measures.^16^^,^^28^ In an additional pooled model, we assessed the impact of the timing of the first dose on the timing of the third dose. The dependent variable was timing of the third dose and the main independent variable was timing of the first dose.

## Results

Data were analysed for 211 643 children aged 12–60 months who had valid records of date of birth and date of mother’s interview. The median survey year was 2012 (interquartile range, IQR: 2010 to 2013). Reported vaccination dates were almost all complete and valid. Overall, vaccination cards were available for 123 679 (weighted count) of the children aged 12–60 months.

At the time of the surveys, 24 countries used the three-dose standard schedule for hepatitis B vaccine (doses at 6, 10 and 14 weeks), four countries vaccinated at 9, 17 and 26 weeks and the remaining countries used other three-dose schedules, some of which included an extra dose at birth, i.e. four doses in total (Table 1). Thirteen countries reported a vaccine dose at birth; eight included a birth dose in their three-dose schedule and five used a four-dose schedule. Combination vaccine, mostly a pentavalent vaccine, was used in 29 countries, while monovalent vaccine was used in 18 countries.

**Fig. 1:**
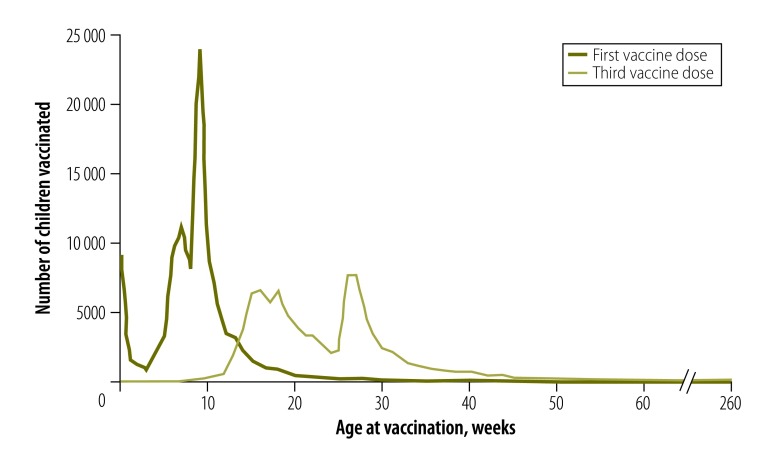
Age at administration of first and third doses of hepatitis B vaccine for all vaccination schedules for children aged 12–60 months in all 47 countries

Notes: Data were extracted from the most recent demographic and health survey in each country (survey year range: 2005–2014). Dates of vaccination were based on vaccination card dates only. Total number of children (weighted counts) were 108 626 (first dose) and 101 542 (third dose).

Fig. 1 shows the pooled distribution of ages at vaccination for 108 626 (first dose) and 101 542 (third dose) children aged 12–60 months at the time of the mother’s interview, using data from vaccination cards only. Both the first and third doses had peak numbers of children vaccinated around the recommended target ages, followed by tails to the right, indicating delays in vaccination. The different peaks in the distributions of first and third doses reflect the diverse immunization schedules and recommended target ages for these doses across the 47 countries.

**Fig. 2:**
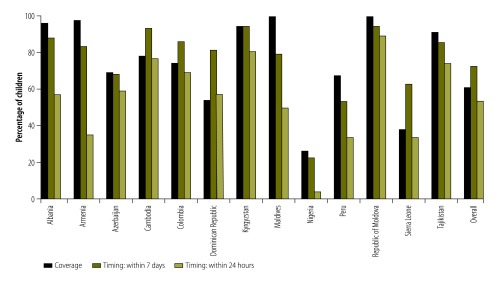
Coverage and timing of birth dose of hepatitis B vaccine for children aged 12–60 months in 13 countries with national vaccination schedules including a vaccine dose at birth

Notes: Data were extracted from the most recent demographic and health survey in each country (survey year range: 2005–2014).

Notes: Coverage is the percentage of children receiving the birth dose of vaccine based on vaccination card data (vaccination dates recorded or vaccination marked without date of administration). Timing of vaccination is the percentage of children receiving the birth vaccine dose, based on two cut-offs: within 7 days of birth and within 24 hours of birth. Denominators are those in Table 2 and Table 5 for countries with a three-dose schedule and a birth-dose vaccine. Denominators for countries with a birth-dose vaccine in a four-dose schedule, for coverage and timing respectively, were as follows: Cambodia: 2604, 2009; Colombia: 9344, 6860; Dominican Republic: 2553, 1372; Peru: 5209, 5165; Sierra Leone: 2560, 943. Dates of vaccination were based on observations with available vaccination dates recorded on vaccination cards.

Coverage of the birth dose ranged from 26% to 99% of children across the 13 countries using this dose. The percentage of children receiving birth-dose vaccinations on time ranged from 23% to 94% across countries (Fig. 2). The proportion of timely vaccinations was lower when we defined the birth dose as administered within 24 hours rather than within 7 days of birth.

### Vaccination coverage

Coverage for all doses, and for complete coverage varied greatly, even across countries following the same vaccination schedule and vaccine type (Table 2, available at http://www.who.int/bulletin/volumes/95/3/16.178822). For example, complete coverage for countries using the 6-, 10-, and 14-week schedule ranged from 13% in Mali to 93% in Swaziland. Overall, we recorded a drop in coverage in particular of the third dose compared to the first dose, irrespective of the vaccination schedule and vaccine type in use. This was particularly prominent in some countries, such as Azerbaijan (where coverage dropped from 69% to 48%) and Côte d’Ivoire (from 74% to 58%).

**Table 2:** Coverage of doses of hepatitis B vaccine for children aged 12–60 months in 47 low- and middle-income countries based on vaccination cards, by national hepatitis B vaccination schedule

Vaccination schedule^a^ and vaccine type	Country	First dose			Second dose			Third dose			Complete^b^	
		No. of children with vaccination data	No. (%) vaccinated		No. of children with vaccination data	No. (%) vaccinated		No. of children with vaccination data	No. (%) vaccinated		No. of children with vaccination data	No. (%) vaccinated
**Weeks 0, 4, 13**												
Monovalent	Maldives	2 073	2 042 (99)		2 079	2 041 (98)		2 078	2 037 (98)		2 078	2 034 (98)
**Weeks 0, 4, 26**												
Monovalent	Republic of Moldova	1 045	1 040 (100)		1 086	1 068 (98)		1 095	1 062 (97)		1 057	1 025 (97)
**Weeks 0, 6, 14**												
Monovalent	Nigeria	14 623	3 735 (26)		15 223	3 442 (23)		16 133	3 113 (19)		15 922	2 880 (18)
**Weeks 0, 6, 26 **												
Monovalent	Armenia	1 041	1 016 (98)		1 042	979 (94)		1 049	943 (90)		1 048	943 (90)
**Weeks 0, 9, 17**												
Monovalent	Azerbaijan	1 106	760 (69)		1 229	721 (65)		1 300	622 (48)		1 292	567 (44)
Monovalent	Tajikistan	3 323	3 026 (91)		2 953	2 780 (94)		3 025	2 750 (91)		3 180	2 740 (86)
**Weeks 0, 9, 22**												
Monovalent	Kyrgyzstan	2 393	2 247 (94)		2 207	2 136 (97)		2 268	2 055 (91)		2 330	2 036 (87)
**Weeks 0, 9, 26**												
Monovalent	Albania	848	813 (96)		886	814 (92)		925	772 (83)		913	759 (83)
**Weeks 4, 8, 12**												
Tetravalent	United Republic of Tanzania	4 424	3 394 (77)		4 465	3 351 (75)		4 565	3 247 (71)		4 556	3 230 (71)
Pentavalent	Uganda	905	809 (89)		957	770 (80)		1 107	710 (64)		1 106	695 (63)
**Weeks 6, 10, 14**												
Monovalent	Bangladesh	3 790	3 592 (95)		3 817	3 532 (93)		3 881	3 446 (89)		3 873	3 438 (89)
Monovalent	Cameroon	2 457	1 751 (71)		2 618	1 697 (65)		2 856	1 614 (57)		2 861	1 606 (56)
Monovalent	Gabon	1 732	802 (46)		1 828	741 (41)		1 870	630 (34)		1 886	624 (33)
Monovalent	Lesotho	877	747 (85)		849	696 (82)		852	657 (77)		876	642 (73)
Monovalent	Pakistan	1 636	561 (34)		1 704	527 (31)		1 904	513 (27)		1 903	513 (27)
Monovalent	Swaziland	1 395	1 348 (97)		1 400	1 335 (95)		1 422	1 318 (93)		1 422	1 317 (93)
Monovalent	Timor-Leste	4 165	2 107 (51)		4 416	2 068 (47)		4 836	2 030 (42)		4 806	2 004 (42)
Bivalent	Benin	6 390	2 355 (37)		6 385	2 263 (35)		6 382	2 146 (34)		6 378	2 122 (33)
Tetravalent	Madagascar	2 643	2 030 (77)		2 748	1 994 (73)		2 919	1 924 (66)		2 886	1 888 (65)
Tetravalent	Mozambique	6 249	5 539 (89)		6 326	5 330 (84)		6 598	5 034 (76)		6 604	5 007 (76)
Pentavalent	Burundi	1 418	1377 (97)		1 418	1 354 (95)		1 457	1 336 (92)		1 457	1 332 (91)
Pentavalent	Cambodia^c^	2 646	2 443 (92)		2 702	2 382 (88)		2 798	2 287 (82)		2 701	1 872 (69)
Pentavalent	Comoros	1 509	1 090 (72)		1 556	1 065 (68)		1 702	1 037 (61)		1 675	1 007 (60)
Pentavalent	Democratic Republic of the Congo	2 246	1 017 (45)		2 590	962 (37)		3 305	894 (27)		3 301	888 (27)
Pentavalent	Côte d’Ivoire	1 846	1 364 (74)		1 893	1 273 (67)		1 929	1 122 (58)		1 917	1 114 (58)
Pentavalent	Ghana	1 672	1 588 (95)		1 716	1 580 (92)		1 829	1 541 (84)		1 819	1 526 (84)
Pentavalent	Kenya	2 647	2 430 (92)		2 733	2 403 (88)		2 892	2 321 (80)		2 851	2 276 (80)
Pentavalent	Liberia	1 079	863 (80)		1 164	812 (70)		1 411	751 (53)		1 405	745 (53)
Pentavalent	Malawi	2 547	2 395 (94)		2 599	2 404 (92)		2 665	2 367 (89)		2 642	2 331 (88)
Pentavalent	Mali	3 627	498 (14)		3 623	479 (13)		3 629	464 (13)		3 629	454 (13)
Pentavalent	Namibia	893	855 (96)		934	849 (91)		971	835 (86)		969	834 (86)
Pentavalent	Niger	1 504	1 155 (77)		1 560	1 113 (71)		1 693	1 066 (63)		1 694	1 062 (63)
Pentavalent	Rwanda	3 030	2 417 (80)		3 044	2 406 (79)		3 063	2 375 (78)		3 056	2 366 (77)
Pentavalent	Senegal	2 472	2 290 (93)		2 468	2 224 (90)		2 472	2 108 (85)		2 467	2 098 (85)
Pentavalent	Sierra Leone^c^	2 325	2 087 (90)		2 397	2 040 (85)		2 666	1 909 (72)		2 521	882 (35)
Pentavalent	Zambia	6 872	6 468 (94)		6 917	6 307 (91)		7 133	6 021 (84)		7 105	5 929 (83)
**Weeks 9, 13, 17**												
Monovalent	Jordan	3 645	3 620 (99)		3 642	3 584 (98)		3 646	3 567 (98)		3 647	3 558 (98)
Pentavalent	Congo	1 684	1 170 (69)		1 841	1 142 (62)		2 128	1 026 (48)		2 118	1 017 (48)
Pentavalent	Burkina Faso	3 823	3 450 (90)		3 845	3 399 (88)		3 945	3 352 (85)		3 936	3 341 (85)
**Weeks 9, 17, 26**												
Monovalent	Egypt	4 875	4 722 (97)		4 655	4 424 (95)		4 663	4 214 (90)		4 559	4 083 (90)
Monovalent	Colombia^c^	9 036	8 472 (94)		9 101	8 355 (92)		10 189	8 199 (80)		9 910	6 576 (66)
Pentavalent	Bolivia (Plurinational State of)	4 846	4 668 (96)		4 955	4 546 (92)		5 126	4 338 (85)		5 109	4 316 (84)
Pentavalent	Dominican Republic^c^	1 797	1 441 (80)		1 824	1 338 (73)		1 997	1 228 (61)		2 039	1 018 (50)
Pentavalent	Guyana	1 149	1 044 (91)		1 170	1 049 (90)		1 198	1 018 (85)		1 183	1 004 (85)
Pentavalent	Honduras	6 561	6 521 (99)		6 581	6 486 (99)		6 631	6 448 (97)		6 563	6 369 (97)
Pentavalent	Peru^c^	5 576	4 260 (76)		5 727	4 190 (73)		5 962	4 080 (68)		5 888	2 926 (50)
**Weeks 13, 17, 22**												
Pentavalent	Zimbabwe	2 503	1 842 (74)		2 559	1 777 (69)		2 654	1 682 (63)		2 660	1 661 (62)
**Overall (weighted counts)**	N/A	146 943	111 261 (76)		149 432	108 229 (72)		156 819	104 209 (66)		155 798	98 655 (63)

N/A: not applicable.

^a^ Schedule is the target weeks after birth to administer the first, second and third doses of vaccine.

^b^ Vaccination coverage was categorized as complete if the child was recorded as fully immunized with at least three doses of monovalent or combination hepatitis B vaccine. Incomplete coverage was if any of the recommended doses was recorded as 0 (not given), irrespective of whether other doses were missing response items; for instance, if dose 1 and 2 were missing but dose 3 was recorded as 0 we considered the individual as incompletely vaccinated.

^c^ Vaccination schedule in these countries includes a birth dose of hepatitis B vaccine (monovalent), i.e. four doses in total.

Notes: Data were extracted from the most recent demographic and health survey in each country (survey year range: 2005–2014). Denominators are weighted counts of the number of children and are based on children with vaccination dates or vaccinations marked as administered in the vaccination card but without dates. Denominators for individual vaccine doses vary due to the number of observations (children) reporting specific doses as not received and the number of children for whom doses were reported as received.

### Vaccination delays

We observed a substantial variation in delays in receipt of the first and third doses across countries having the same vaccination schedule and vaccine type (Table 3). We noted a drop in timely vaccinations between the first and third doses, irrespective of the vaccination schedule and vaccine type in use.

**Table 3:** Time delays in the receipt of doses of hepatitis B vaccine for children aged 12–60 months in 47 countries, by national hepatitis B vaccination schedule

Vaccination schedule^a^ and vaccine type	Country	First dose			Third dose	
		No. of children vaccinated	No. (%) with delayed vaccination		No. of children vaccinated	No. (%) with delayed vaccination
**Weeks 0, 4, 13**						
Monovalent	Maldives	2 042	427 (21)		2 036	1 868 (92)
**Weeks 0, 4, 26**						
Monovalent	Republic of Moldova	1 040	66 (6)		1 062	355 (33)
**Weeks 0, 6, 14**						
Monovalent	Nigeria	3 661	2 823 (77)		3 043	1 615 (53)
**Weeks 0, 6, 26**						
Monovalent	Armenia	1 016	170 (17)		943	554 (59)
**Weeks 0, 9, 17**						
Monovalent	Azerbaijan	760	244 (32)		622	279 (45)
Monovalent	Tajikistan	2 981	433 (15)		2 750	545 (20)
**Weeks 0, 9, 22**						
Monovalent	Kyrgyzstan	2 244	125 (6)		2 054	348 (17)
**Weeks 0, 9, 26**						
Monovalent	Albania	798	99 (12)		758	96 (13)
**Weeks 4, 8, 12**						
Tetravalent	United Republic of Tanzania	3 367	996 (30)		3 223	1 868 (58)
Pentavalent	Uganda	801	371 (46)		700	528 (75)
**Weeks 6, 10, 14**						
Monovalent	Bangladesh	3 583	818 (23)		3 428	1 792 (52)
Monovalent	Cameroon	1 745	366 (21)		1 607	641 (40)
Monovalent	Gabon	793	211 (27)		627	320 (51)
Monovalent	Lesotho	739	115 (16)		643	266 (41)
Monovalent	Pakistan	560	185 (33)		508	322 (63)
Monovalent	Swaziland	1 347	94 (7)		1 315	337 (26)
Monovalent	Timor-Leste	1 971	740 (38)		1 853	1 112 (60)
Bivalent	Benin	2 076	398 (19)		1 877	879 (47)
Tetravalent	Madagascar	1 993	524 (26)		1 891	882 (47)
Tetravalent	Mozambique	5 282	2 361 (45)		4 764	3 586 (75)
Pentavalent	Burundi	1 335	180 (13)		1 298	517 (40)
Pentavalent	Cambodia^b^	2 443	368 (15)		2 286	850 (37)
Pentavalent	Comoros	1 088	255 (23)		1 032	537 (52)
Pentavalent	Côte d’Ivoire	1 363	396 (29)		1 120	647 (58)
Pentavalent	Democratic Republic of the Congo	914	255 (28)		780	337 (43)
Pentavalent	Ghana	1 587	220 (14)		1 539	579 (38)
Pentavalent	Kenya	2 413	451 (19)		2 302	804 (35)
Pentavalent	Liberia	862	256 (30)		749	461 (61)
Pentavalent	Malawi	2 341	664 (28)		2 309	1 327 (57)
Pentavalent	Mali	309	127 (41)		275	188 (68)
Pentavalent	Namibia	814	69 (8)		796	173 (22)
Pentavalent	Niger	1 148	400 (35)		1 062	707 (67)
Pentavalent	Rwanda	2 386	167 (7)		2 351	569 (24)
Pentavalent	Senegal	2 277	617 (27)		2 084	1 154 (55)
Pentavalent	Sierra Leone^b^	2 072	555 (27)		1 891	1 168 (62)
Pentavalent	Zambia	6 136	1 883 (31)		5 697	3 438 (60)
**Weeks 9, 13, 17**						
Monovalent	Jordan	3 598	381 (11)		3 523	1 264 (36)
Pentavalent	Congo	1 155	161 (14)		1 014	315 (31)
Pentavalent	Burkina Faso	3 447	502 (15)		3 350	1 188 (35)
**Weeks 9, 17, 26**						
Monovalent	Egypt	4 612	220 (5)		4 093	474 (12)
Monovalent	Colombia^b^	8 431	1 194 (14)		8 161	2 510 (31)
Pentavalent	Bolivia (Plurinational State of)	4 631	1 112 (24)		4 292	1 849 (43)
Pentavalent	Guyana	1 044	202 (19)		1 018	416 (41)
Pentavalent	Honduras	6 516	464 (7)		6 445	1 673 (26)
Pentavalent	Peru^b^	4 225	453 (11)		4 065	1 251 (31)
**Weeks 13, 17, 22**						
Pentavalent	Zimbabwe	1 246	341 (27)		1 082	574 (53)
**Overall (weighted counts)**	N/A	108 626	23 626 (22)		101 542	43 548 (43)

N/A: not applicable.

^a^ Schedule is the target weeks after birth to administer the first, second and third doses of vaccine.

^b^ Vaccination schedule in these countries includes a birth dose of hepatitis B vaccine (monovalent), i.e. four doses in total.

Notes: Data were extracted from the most recent demographic and health survey in each country (survey year range: 2005–2014). The results are based on children for whom vaccination dates were available (recorded on vaccination cards). We included children who received vaccinations before the recommended age (early vaccinations) in the denominator. Delayed vaccination was defined as a vaccine dose received more than 4 weeks after the target week in the national vaccination schedule. Estimates of early vaccinations are not shown in the table. The following countries reported > 10% of children vaccinated before the recommended age for the first dose: Burkina Faso (23%), Cameroon (12%), Congo (16%), Democratic Republic of the Congo (14%), Egypt (17%), Guyana (13%), Madagascar (11%), Mali (11%), Sierra Leone (20%) and Timor-Leste (16%). The following countries reported > 10% of children vaccinated before the recommended age for the third dose: Azerbaijan (50%), Plurinational State of Bolivia (12%), Colombia (12%), Kyrgyzstan (60%), Nigeria (12%) and Tajikistan (56%).

**Fig. 3:**
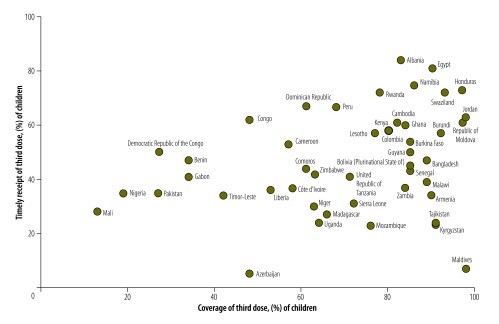
Scatter plot of country-specific coverage and timing of third dose of hepatitis B vaccine for children aged 12–60 months in 47 countries

Notes: Correlation between vaccination timing and coverage, Spearman rho = 0.28, *P* = 0.05. Data were extracted from the most recent demographic and health survey in each country (survey year range: 2005–2014). Coverage is the percentage of children receiving the third dose of vaccine based on vaccination card data (vaccination dates recorded or vaccination marked without date of administration). Timely receipt of vaccination is the percentage of children receiving the third dose within 4 weeks of the target age (weeks) of the national vaccination schedule. Denominators are those in Table 2 and Table 3. Dates of vaccination were based on observations with available vaccination dates recorded on vaccination cards. We included children vaccinated before the recommended age (early vaccinations) in the denominator when calculating delayed and timely vaccination rates. Estimates of early vaccinations are not shown in the figure. The following countries reported > 10% children vaccinated before the recommended age for the third dose: Azerbaijan (50%), Plurinational State of Bolivia (12%), Colombia (12%), Kyrgyzstan (60%), Nigeria (12%) and Tajikistan (56%).

For the 47 countries overall, the median of the median delays for the first vaccine dose was 1.0 week, and the 75th percentile was 3.6 weeks, i.e. in 25% of the countries the median delay was more than 3.6 weeks. For the third dose, the delays were more than twice as long (Table 4). The country-specific distribution of ages at vaccination had long tails, and delays at the 90th percentile were at least twice as long as the 75th percentile (Table 5, available at http://www.who.int/bulletin/volumes/95/3/16.178822). Overall, WHO African Region countries tended to have lower vaccination coverage and poorer timing compared with countries in the Americas and Europe. Delays were recorded even in countries with high coverage, such as Bangladesh and Burkina Faso. We found a weak positive correlation (Spearman rho = 0.28; *P* = 0.05) between vaccination timing and coverage. Fig. 3 shows the timing and the corresponding coverage of the third vaccine dose for each of the 47 countries, using data from vaccination cards.

**Table 4:** Time delays in the receipt of doses of hepatitis B vaccine for children aged 12–60 months across 47 countries

Percentiles	First dose delay percentiles, weeks				Third dose delay percentiles, weeks		
	25th	50th	75th		25th	50th	75th
25th	0.0	0.4	1.8		0.7	2.4	6.1
50th (median)	0.3	1.0	3.6		1.4	3.7	9.3
75th	0.6	2.0	5.0		2.4	5.7	13.2

Notes: Total number of children (weighted counts) were 108 626 (first dose) and 101 542 (third dose). Data were extracted from the most recent demographic and health survey in each country (survey year range: 2005–2014). Delayed vaccination was a vaccine dose received more than 4 weeks after the target week in the national vaccination schedule.

**Table 5:** Time delays, in percentiles, in the receipt of doses of hepatitis B vaccine for children aged 12–60 months in 47 countries, by national hepatitis B vaccination schedule

Vaccination schedule^a^ and vaccine type	Country or median for vaccination schedule	First dose						Third dose				
		No. of children vaccinated	Delay percentiles, weeks					No. of children vaccinated	Delay percentiles, weeks			
			25th	50th	75th	IQR			25th	50th	75th	IQR
**Weeks 0, 4, 13**												
Monovalent	Maldives	2042	0.1	0.3	1.0	0.9		2036	5.9	7.9	11.9	6.0
**Weeks 0, 4, 26**												
Monovalent	Republic of Moldova	1040	0.0	0.0	0.1	0.1		1062	0.6	2.3	5.6	5.0
**Weeks 0, 6, 14**												
Monovalent	Nigeria	3661	1.7	4.7	9.4	7.7		3043	1.0	5.4	14.7	13.7
**Weeks 0, 6, 26**												
Monovalent	Armenia	1 016	0.1	0.3	0.6	0.4		943	2.0	6.1	13.0	11.0
**Weeks 0, 9, 17**												
Monovalent	Azerbaijan	760	0.0	0.0	4.4	4.4		622	0.9	3.1	10.1	9.3
Monovalent	Tajikistan	2981	0.0	0.0	0.3	0.3		2750	−3.3	−1.1	3.0	6.3
N/A	Median	1541	0.0	0.0	2.4	2.4		1499	−1.2	1.0	6.6	7.8
**Weeks 0, 9, 22**												
Monovalent	Kyrgyzstan	2244	0.0	0.1	0.1	0.1		2054	−6.1	−3.3	2.1	8.3
**Weeks 0, 9, 26 **												
Monovalent	Albania	798	0.1	0.1	0.3	0.2		758	0.4	1.1	2.7	2.3
**Weeks 4, 8, 12**												
Tetravalent	United Republic of Tanzania	3367	0.9	2.3	5.1	4.3		3223	2.4	5.6	11.9	9.4
Pentavalent	Uganda	801	2.7	4.1	7.9	5.2		700	4.6	8.6	17.7	13.1
N/A	Median	2084	1.8	3.2	6.5	4.7		1962	3.5	7.1	14.8	11.3
**Weeks 6, 10, 14**												
Monovalent	Bangladesh	3583	1.0	2.6	4.3	3.3		3428	2.6	4.7	8.7	6.1
Monovalent	Cameroon	1745	0.3	1.1	3.9	3.6		1607	1.1	3.1	7.7	6.6
Monovalent	Gabon	793	0.4	1.1	5.1	4.7		627	1.9	4.7	13.0	11.1
Monovalent	Lesotho	739	0.4	1.1	2.9	2.4		643	2.0	3.7	7.9	5.9
Monovalent	Pakistan	560	1.0	2.7	6.1	5.1		508	3.1	5.9	13.4	10.3
Monovalent	Swaziland	1347	0.1	0.4	1.3	1.2		1315	0.7	1.7	4.6	3.9
Monovalent	Timor-Leste	1971	0.4	3.0	7.6	7.1		1853	2.6	6.1	12.9	10.3
Bivalent	Benin	2076	0.1	1.0	3.4	3.3		1877	1.3	4.0	9.4	8.1
Tetravalent	Madagascar	1993	0.4	2.0	4.7	4.3		1891	1.9	4.0	9.3	7.4
Tetravalent	Mozambique	5282	2.7	4.0	7.7	5.0		4764	4.6	9.3	19.3	14.7
Pentavalent	Burundi	1335	0.6	1.1	2.6	2.0		1298	2.0	3.4	6.6	4.6
Pentavalent	Cambodia^b^	2443	0.6	0.9	2.7	2.1		2286	1.6	3.0	6.9	5.3
Pentavalent	Comoros	1088	0.4	1.1	4.0	3.6		1032	2.0	5.0	13.6	11.6
Pentavalent	Côte d’Ivoire	1363	0.6	2.0	5.6	5.0		1120	2.9	5.9	14.3	11.4
Pentavalent	Democratic Republic of the Congo	914	0.3	1.7	5.0	4.7		780	1.3	3.7	9.7	8.4
Pentavalent	Ghana	1587	0.3	1.1	3.1	2.9		1539	1.4	3.3	6.6	5.1
Pentavalent	Kenya	2413	0.1	1.0	3.4	3.3		2302	0.9	2.6	6.6	5.7
Pentavalent	Liberia	862	0.4	1.7	5.0	4.6		749	2.1	6.4	17.0	14.9
Pentavalent	Malawi	2341	0.7	2.4	5.0	4.3		2309	2.6	5.6	11.0	8.4
Pentavalent	Mali	309	0.7	2.9	8.3	7.6		275	3.9	7.3	19.4	15.6
Pentavalent	Namibia	814	0.0	0.4	1.0	1.0		796	0.6	1.4	3.9	3.3
Pentavalent	Niger	1148	0.6	2.6	7.0	6.4		1062	3.1	7.3	16.6	13.4
Pentavalent	Rwanda	2386	0.4	1.0	2.3	1.9		2351	1.1	2.4	4.4	3.3
Pentavalent	Senegal	2277	0.6	1.7	4.7	4.1		2084	2.1	5.3	11.1	9.0
Pentavalent	Sierra Leone^b^	2072	0.0	1.3	4.9	4.9		1891	2.4	7.3	17.0	14.6
Pentavalent	Zambia	6136	0.4	2.0	5.4	5.0		5697	2.4	6.3	15.0	12.6
N/A	Median	1587	0.4	1.5	4.7	4.2		1573	2.0	4.7	10.4	8.4
**Weeks 9, 13, 17**												
Monovalent	Jordan	3598	0.0	0.7	2.1	2.1		3523	1.6	3.1	6.1	4.6
Pentavalent	Congo	1155	−0.1	0.4	2.7	2.9		1014	0.7	2.1	5.6	4.9
Pentavalent	Burkina Faso	3447	−0.4	0.4	2.4	2.9		3350	0.7	2.7	6.4	5.7
N/A	Median	3447	−0.1	0.4	2.4	2.9		3350	0.7	2.7	6.1	4.9
**Weeks 9, 17, 26**												
Monovalent	Egypt	4612	−0.3	0.1	0.9	1.2		4093	0.3	0.9	2.3	2.0
Monovalent	Colombia^b^	8431	−0.1	0.3	2.1	2.3		8161	0.4	1.7	6.1	5.7
Pentavalent	Bolivia (Plurinational State of)	4631	−0.1	1.0	4.3	4.4		4292	0.4	3.0	10.0	9.6
Pentavalent	Dominican Republic^b^	1434	−0.1	0.1	1.4	1.6		1224	1.0	2.1	6.0	5.0
Pentavalent	Guyana	1044	−0.1	1.0	3.6	3.7		1018	1.1	3.3	8.1	7.0
Pentavalent	Honduras	6516	−0.3	0.0	1.0	1.3		6445	0.6	1.7	4.7	4.1
Pentavalent	Peru^b^	4225	−0.3	0.0	1.4	1.7		4065	0.3	1.7	5.7	5.4
N/A	Median	4612	−0.1	0.1	1.4	1.7		4093	0.4	1.7	6.0	5.4
**Weeks 13, 17, 22**												
Pentavalent	Zimbabwe	1246	0.3	1.7	4.9	4.6		1082	1.1	5.3	14.0	12.9

IQR: interquartile range; N/A: not applicable.

^a^ Schedule is the target week after birth to administer the first, second and third doses of vaccine.

^b^ Vaccination schedule in these countries includes a birth dose of hepatitis B vaccine (monovalent), i.e. four doses in total.

Notes: Data were extracted from the most recent demographic and health survey (survey year range: 2005–2014) in each country. Denominators are weighted. Delayed vaccination was vaccine dose received more than 4 weeks after the target week in the national vaccination schedule. Negative values indicate vaccination before the recommended target week; 0.0 indicates no delays.

Table 6 (available at http://www.who.int/bulletin/volumes/95/3/16.178822) shows the descriptive statistics for the pooled weighted sample used in the regression models. Table 7 shows pooled multivariable regression models for delays in the first and third doses. After adjusting for covariates, delays in the first dose for vaccination schedules starting at 6 weeks of age (aOR: 0.81; 95% CI: 0.75 to 0.88) and at 9 weeks of age (aOR: 0.50; 95% CI: 0.46 to 0.53) were lower than for vaccination schedules with a birth dose. Vaccination schedules starting at 4 weeks and at 13 weeks of age tended to have higher odds of delays. Combination vaccines tended to have lower odds of delays in the first dose than did the monovalent vaccine (aOR: 0.76; 95% CI: 0.71 to 0.81). In a separate pooled model, when controlling for the timing of the receipt of the first dose, we observed higher odds of delays in the third dose if the first dose was delayed than if it was on time (aOR: 22.89; 95% CI: 20.99 to 24.97).

**Table 6:** Descriptive characteristics of children aged 12–60 months included in the study on the association between vaccination schedules (vaccine type) and hepatitis B vaccination timing in 47 countries

Characteristic	No. (%) of children
**Child’s sex**	
Male	105 351 (51)
Female	102 095 (49)
**Residence**	
Urban	75 470 (36)
Rural	131 976 (64)
**Birth order**	
First child	53 614 (26)
Second or higher child	153 832 (74)
**Place of delivery**	
Home	64 666 (31)
Institution	138 963 (67)
Missing data	3817 (2)
**Mother’s education**	
None	55 907 (27)
Primary	67 851 (33)
Secondary or higher	83 642 (40)
Missing	45 (< 1)
**Mother’s marital status**	
Unmarried	55 614 (27)
Married	151 832 (73)
**Wealth index^a^**	
Poorest	46 606 (22)
Poor	44 791 (22)
Medium	42 917 (21)
Rich	39 492 (19)
Richest	33 641 (16)
**Family size, mean (95% CI)**	6.62 (6.57 to 6.67)
**Country income level^b^**	
Low	68 224 (33)
Lower-middle	103 415 (50)
Upper-middle	35 807 (17)
**Total (weighted)**	207 446 (100)
**Population size (unweighted)**	211 643

CI: confidence interval.

^a^ Wealth index as an indicator of economic status of the household, categorized into five quintiles ranging from the poorest 20% to the richest 20%.

^b^ Country income level as per the World Bank.^22^

Notes: Missing observations (non-responses) were excluded from the analysis. Numbers are weighted counts.

**Table 7:** Multivariable pooled regression analysis for the association between vaccination schedule and vaccine type on hepatitis B vaccination timing among children aged 12–60 months in 47 countries

Variable	First dose			Third dose		
	No. of children vaccinated^a^	No. of children with delays	aOR (95% CI)	No. of children vaccinated^a^	No. of children with delays	aOR (95% CI)
**Vaccination schedule start week**						
≤ 1	14 437	4 353	Ref.	9 565	5 602	Ref.
4	3 972	1 353	0.91 (0.80 to 1.03)	3 810	2 355	1.14 (1.00 to 1.30)
6	44 647	12 525	0.81 (0.75 to 0.88)	43 932	23 336	0.97 (0.91 to 1.03)
9	29 151	4 482	0.45 (0.41 to 0.50)	33 273	10 688	0.50 (0.46 to 0.53)
13	791	338	1.11 (0.92 to 1.34)	1 016	565	1.21 (1.03 to 1.42)
**Vaccine type**						
Monovalent	37 763	8 305	Ref.	32 297	14 007	Ref.
Combination	60 055	14 746	0.76 (0.71 to 0.81)	59 299	28 538	0.99 (0.94 to 1.05)

aOR: adjusted odds ratio; CI: confidence interval; Ref.: reference category.

^a^ The number of children included in the analyses were adjusted for the covariates stated below.

Notes: Data were extracted from the most recent demographic and health survey (DHS) in each country (survey year range: 2005–2014). Total number of children (weighted counts) were 97 818 (first dose) and 91 596 (third dose). Total observations were 100 167 (first dose) and 93 807 (third dose). Denominators vary across variables because of item non-response. Model was adjusted for child’s age (yearly increments), sex, residence (urban versus rural), birth order of the child (1 versus > 1), mother’s age (yearly increments), mother’s marital status (married versus unmarried), mother’s education (none, primary, secondary and higher), birth place (home versus institutional), household wealth index (5 quintiles of wealth; poorest, poor, medium, rich, richest), family size (increments per member), country income level as per the World Bank (categorized as low income, lower-middle income and upper-middle income;^22^ and survey year. The variance inflation factors for the multivariable models were 1.06 for first dose (delayed) and 1.09 for third dose (delayed), respectively, indicating the absence of multicollinearity among explanatory variables.

## Discussion

Our analysis of survey data from 47 low- and middle-income countries, inhabited by around 1.2 billion people,^29^ showed a wide variation in hepatitis B vaccination coverage and timing across countries. The results highlight differences in vaccination implementation, and in adherence to national immunization schedules. This may reflect differences in barriers to immunization, in inequities in health-care delivery and access, as upper-middle-income countries tended to have better coverage and timing than lower-middle and low-income countries. Most countries had fairly high coverage (> 80%), in particular for the first dose, and delivered vaccines on time. Although this finding is encouraging, in most countries coverage decreased and delays increased with subsequent doses, irrespective of a country’s specific vaccination schedule. Crucially, vaccination coverage was low (< 50%) and vaccinations were delayed in populous countries that are highly endemic for HBV infection, such as Nigeria.

Despite WHO recommendations on hepatitis B vaccination within 24 hours,^8^ only 13 countries in our analysis reported using a birth dose, with wide variations in its coverage and timing. Due to existing sociocultural, financial, infrastructural and logistic constraints on vaccine delivery, many countries do not require the birth dose to be strictly administered within 24 hours of birth.^26^^,^^30^ A major challenge, particularly in highly endemic, resource-poor countries with a high proportion of home deliveries, is ensuring the timely administration of the birth dose to every child irrespective of where he or she is born.^30^^,^^31^

Most countries where the HBV epidemic is concentrated have adopted the three-dose combination vaccine delivered at 6, 10 and 14 weeks.^30^ Our analysis gave some indication that vaccination delays were lower with vaccination schedules starting at 6 or 9 weeks of age compared with those starting at or before 1 week of age, and with combination vaccines as compared with monovalent vaccines. This might be attributable to increased compliance by vaccine recipients due to the reduced number of injections and fewer visits required to health-care facilities.^32^ That said, administering combination vaccinations at 6 or 9 weeks of age, while cost-effective and simple, cannot prevent vertical and early horizontal transmission.^30^

It has been suggested that, due to the predominantly horizontal routes of HBV transmission in Africa, the benefit of implementing a birth dose would not justify the necessary financial, human resource and infrastructure investments.^33^ This is based on the premise that perinatal transmission is not a major factor in HBV transmission due to the lower prevalence of hepatitis B e-antigen (HBeAg) positivity in pregnant women in Africa. However, studies suggest that up to 38% of pregnant African women with chronic HBV are positive for HBeAg and hence at high risk of transmitting infection to their infants.^34^^–^^36^ Data on the epidemiology of HBV, particularly transmission routes,^30^ and on the benefits of birth-dose vaccination are scarce in Africa.^37^ Nevertheless, in our view, the benefits of giving a birth dose in the African setting deserve consideration, due to the high burden of HBV infection^2^ and the known high risk of infection and chronicity associated with perinatal and early horizontal infections. From a policy perspective it is important to examine current country-level modes of HBV transmission in tandem with existing vaccination schedules so that recommendations can be adapted to existing disease transmission patterns.

We found lower compliance with national schedules for the second and third vaccine doses and a weak correlation of timing with coverage. This implies that even in countries with relatively high coverage, children who achieve complete vaccination may spend a considerable period of time with no or incomplete protection. This is particularly concerning in countries with a high burden of infection.^3^

Our analysis also indicates that the third dose of vaccine is more likely to be delayed among those who received a delayed first dose. This suggests that prioritizing timely first vaccinations could result in the timely receipt of successive doses^38^ and avert delays that would require catch-up regimens. Given the existent challenges in providing hepatitis B vaccination in resource-poor settings, catch-up regimens might decrease the likelihood of the timely completion of the hepatitis B vaccination series.^38^^,^^39^ This underscores the need to incorporate the monitoring of vaccination timing, in addition to coverage, into vaccination programmes.

Interrupting transmission routes for HBV warrants comprehensive strategies to prevent mother-to-child transmission and to deliver adequate and timely immunoprophylaxis in newborns^40^ and infants.^41^^,^^42^ In remote, resource-constrained settings, integrating vaccine administration with assisted home deliveries and employing out-of-cold-chain strategies might be possible solutions to improve timely vaccination coverage.^43^^–^^45^ Furthermore, mathematical models, calibrated to country-specific HBV epidemiology might be useful to quantify the burden of infection attributable to delayed vaccinations. In this context, models could be developed to assess the infections and deaths averted by prioritizing timely vaccinations that use alternative vaccination schedules and diverse outreach strategies.

### Limitations

The main limitation of this analysis is related to the available data from DHS. The survey years varied substantially across countries, and therefore caution is warranted when interpreting international comparisons.^20^ Most surveys were fairly recently conducted – the median survey year was 2012– and provide useful insights into the quality (timing) and quantity (coverage) of current hepatitis B vaccination programmes. However, some of the older surveys, notably in the Republic of Moldova and Swaziland, may not reflect the current situation.

The distribution of ages at vaccination are only crude indicators of the timing issue, since each country’s contribution was determined by the size of its survey sample, which varied among countries and did not reflect actual population sizes.

Our coverage estimates vary to some extent from available estimates^46^ due to some aspects of our method: the use of DHS survey data, the age groups included and the reliance on documented vaccinations. Multisurvey prospective data were unavailable for most countries. We could not therefore assess temporal changes in vaccination measures and the effects of changes in vaccination schedules or vaccine types on the studied outcomes. Furthermore, some vaccination schedules included in the analysis were used only by a small number of countries, which impeded any conclusions about the effects of specific schedules. We restricted our analysis to established vaccination schedules. This might lead to underestimates or overestimates depending on the uptake of newer vaccines and schedules by countries. Data on vaccination service providers were not available which might have provided valuable insights into the issue of hepatitis B vaccination timing.

We excluded undocumented vaccinations from the analysis and therefore coverage and delays may be underestimates, since undocumented vaccinations including lost or misplaced vaccination cards were not captured.^19^ Vaccination information was based only on maternal recall in approximately 30% of the observations, with higher figures in some countries (such as the Democratic Republic of the Congo and Nigeria). However, no noteworthy differences in coverage were detected for most countries when we included maternal reports (data are available from the corresponding author).

A disadvantage of cross-sectional studies is the potential for survivor bias. Our analysis did not include deceased children since the included surveys did not record vaccination data for this sub-group. We might have overestimated vaccination measures slightly since it is unlikely that deceased children would have better vaccination parameters than surviving children.^47^ The cross-sectional nature of the data also precluded our drawing causal inferences. Additionally, it is likely that there was residual confounding that was not adjusted for in our models. To enable more in-depth analyses, future surveys need to incorporate sufficiently detailed questions on barriers to immunization, e.g. vaccine availability in the health system, and on parental and provider vaccination practices.

Lastly, the surveyed countries were not randomly sampled. Hence the external validity of the results for other low- and middle-income countries might be limited, particularly for those using different vaccination schedules than those in the current analysis. The available data were primarily from countries in the WHO African, European and Americas Regions, with limited data from the Eastern Mediterranean, South-East Asian and Western Pacific Regions.

## Conclusion

The substantial inequities in the implementation and adherence to national immunization schedules for hepatitis B vaccine underscore the continued need for strengthening immunization systems. Strategies that focus on the timely initiation of hepatitis B immunization might lead to the timely receipt of successive doses and hence improve overall coverage. Our findings indicate that timing should be incorporated as a performance indicator of routine immunization services, as a complement to coverage assessments.
