# Corrigendum

**DOI:** 10.2471/BLT.17.100417

**Published:** 2017-04-01

In Volume 95, Issue 3, March 2017, page 202, Table 3 should have an additional row: 

**Table 3:** Time delays in the receipt of doses of hepatitis B vaccine for children aged 12–60 months in 47 countries, by national hepatitis B vaccination schedule

Vaccination schedule^a^ and vaccine type	Country	First dose			Third dose	
		No. of children vaccinated	No. (%) with delayed vaccination		No. of children vaccinated	No. (%) with delayed vaccination
Pentavalent	Dominican Republic^b^	1 434	167 (12)		1 224	385 (31)

Schweitzer A, Akmatov MK, Krause G. Hepatitis B vaccination timing: results from demographic health surveys in 47 countries. Bull World Health Organ. 2017 Mar 1;95(3):199–209G. http://dx.doi.org/10.2471/BLT.16.178822

